# Supplementary material for: Impact of Low Skeletal Muscle Mass on Long-Term Outcomes in Hepatocellular Carcinoma Treated with Trans-Arterial Radioembolization: A Retrospective Multi-Center Study
Source: Cancers (Basel). 2023 Oct 28;15(21):5195. doi: 10.3390/cancers15215195 (PMC10647696; doi:10.3390/cancers15215195)
Supplement: Supplementary file 1 [file cancers-15-05195-s001.zip › cancers-2656732-supplementary.pdf]

cancers MDPI

Table S1. Details of TARE procedure.

Variables	Total<br>(*n* = 347)	Non sarcopenic<br>(*n* = 221)	Sarcopenic<br>(*n* =126)	*p* value
Hepatopulmonary shunt (%)	6.7 (4.8–9.7)	6.7 (5.0–9.5)	6.6 (4.4–10.3)	0.780
Type of microsphere				
Resin	212 (61.1%)	145 (65.6%)	67 (53.2%)	0.022
Glass	135 (38.9%)	79 (34.4%)	59 (46.8%)	
Administered activity (GBq)	2.5 (1.7–3.4)	2.5 (1.7–3.1)	2.7 (1.6–3.8)	0.210

Variables expressed as median (interquartile range) or n (%); TARE, trans-arterial radioembolization.
